# They (Don’t) Need Us: Functional Indispensability Impacts Perceptions of Representativeness and Commitment When Lower-Status Groups Go Through an Intergroup Merger

**DOI:** 10.3389/fpsyg.2019.02772

**Published:** 2020-01-14

**Authors:** Miriam Rosa, Steffen Giessner, Rita Guerra, Sven Waldzus, Anna-Maria Kersting, Katarina Veličković, Elizabeth C. Collins

**Affiliations:** ^1^Instituto Universitário de Lisboa (ISCTE-IUL), CIS-IUL, Lisbon, Portugal; ^2^Rotterdam School of Management, Erasmus University Rotterdam, Rotterdam, Netherlands; ^3^Department of Psychology, University of Salzburg, Salzburg, Austria; ^4^Department of Psychology, Lund University, Lund, Sweden

**Keywords:** intergroup relations, intergroup change, mergers, lower-status groups, prototypicality, functional indispensability, information processing

## Abstract

Intergroup changes occur often between subgroups who are asymmetric in status (e.g., size, power, prestige), with important consequences for social identification, especially among the members of lower-status groups. Mergers offer an example of such changes, when subgroups (merger partners) merge into a common, superordinate group (post-merger group). Lower-status subgroups frequently perceive they are less represented in the post-merger group, therefore committing less to the changes a merger implies. Five studies offered an intergroup relations’ perspective on mergers (*N*’s = 479, 150, 266, 113, and 229, respectively), examining how functional indispensability (instrumental contribution of the ingroup) positively influences perceptions of representativeness in the post-merger group (relative ingroup prototypicality), which, in turn, affect post-merger identification and, finally, change commitment. Additionally, the role of cognitive information processing (heuristic vs. systematic) on prototypicality was explored. Results suggest that functional indispensability impacts relative ingroup prototypicality (Studies 1–5), and this may be moderated by information processing (Study 2). Moreover, prototypicality and identification with the superordinate post-merged group mediated the effect of functional indispensability on change commitment (Studies 1–3). These findings provide important theoretical insights into prototypicality perceptions held by lower-status merger partners and minority groups in general, by identifying functional indispensability as a source of prototypicality other than relative status. In addition, by proposing a functional approach to the relations between social groups, these findings suggest better practices for managing structural changes, such as combining sources of strategic/functional and identity fit when announcing an intergroup change.

## Introduction

Mergers are a very common phenomenon, occurring in diverse groups (schools, municipalities, corporations, and even countries). Despite being a popular strategy, mergers^1^ create instability among group members from the moment they are announced (van Dick et al., 2006) and elicit negative reactions ultimately affecting the success of these unions (Hogan and Overmyer-Day, 1994; Cartwright, 2006). Mergers involve the integration of at least two subgroups (often called merger partners), one of which is usually of lower pre-merger status than the other, for example, by being smaller or having less power. These lower-status partners often face difficulties in integration, perceiving their pre-merger group as less represented within the post-merger group (Gleibs et al., 2008), and experiencing a lower sense of identity continuity (van Knippenberg et al., 2002). However, some groups are considered for a merger because they might have something valuable to attain a superordinate goal. We frame this as functional indispensability (Guerra et al., 2015) and test it as a predictor of perceived representativeness (relative ingroup prototypicality; Wenzel et al., 2007) in the merged group and also as a potential facilitator of identification and commitment to change in members of lower-status merger partners. Specifically, extending previous research that considered indispensability and prototypicality as parallel processes, we propose that lower-status merger partners can perceive higher relative ingroup prototypicality in the merged group when they consider the ingroup as functionally indispensable to the goals of the post-merged group. We focused on the early stage of a merger, when it is announced, and explored cognitive-motivational aspects (Chaiken and Trope, 1999) influencing representativeness claims. Although this framework can apply to intergroup relations in general, we focus on organizational mergers because they offer an optimal context to approach the role of functional indispensability.

### Identity Fit: The Social Identity Perspective

Being part of a group is important for people’s identity, including membership in organizations (Haslam, 2004). However, for members of merging organizations, a merger threatens one’s organizational identification because the change triggers an insecure social identity context (Tajfel and Turner, 1986). Mergers imply that a pre-merger group is recategorized as part of a higher-order merged group, together with another subgroup with whom it might share very little. This recategorization can trigger “us vs. them” mindsets, inflating identity misfits that are detrimental to merger acceptance/support (Gleibs et al., 2008). Therefore, the acceptance of a merger benefits from merger partners’ members identifying with the superordinate, post-merger group (see Giessner et al., 2012 for an overview). Post-merger identification is more likely if group members see their pre-merger group represented in the post-merger group (Giessner et al., 2006; Gleibs et al., 2008). However, this is particularly difficult to attain for all partners: The larger or economically better-off have higher pre-merger status and are usually more represented (van Leeuwen et al., 2003; Gleibs et al., 2013), creating an asymmetric intergroup context. Sources of intergroup asymmetries can be different [e.g., size, prestige, power (Sachdev and Bourhis, 1985); influence (Turner, 2005)], but they all involve intergroup comparisons on dimensions that are relevant for hierarchical differentiation (Scheepers et al., 2009). Dominance might derive from prestige, from power (Magee and Galinsky, 2008), but in some constellations, these dimensions do not go together [e.g., when a more economically powerful partner acquires a more prestigious one (van Knippenberg et al., 2002)]. For the sake of simplicity, as long as one partner is consensually expected to take the lead upon the other based on intergroup asymmetries, we consider that this partner has a higher pre-merger status.

One consequence of such asymmetries is that the members of the lower-status partner often perceive themselves less represented within the merged group than the higher-status merger partner’s members (van Leeuwen et al., 2003; Gleibs et al., 2013). Consequently, these lower-status partner’s members often identify less with the merged group (Giessner et al., 2016) and are more prone to show detrimental reactions resulting from that low identification [e.g., lack of support for the merger and low commitment with the group (Boen et al., 2006; Amiot et al., 2007; Fischer et al., 2007; Jonas and Mummendey, 2008)].

Lower-status merger partners may support the group changes implied in a merger as far as they can envision to acquire a positive social identity within the post-merger framework. For higher-status merger partners, maintaining positive social identity is unproblematic, as they tend to take their own culture for granted as defining normality, and perceive the other group as deviating from that normality (Haspeslagh and Jemison, 1991; DeRoche, 1997). Efforts to transform the other group like one’s own can follow these perceptions. This has been explained by ingroup projection (Mummendey and Wenzel, 1999): when a group (ingroup) compares with other group(s) [outgroup(s)], they do so based on a higher-order group including all groups under comparison (superordinate category; SC) (Turner et al., 1987), but there is a general tendency to project distinctive ingroup rather than outgroup attributes to that superordinate category. Groups can vary in the extent to which they are prototypical of the superordinate category, and this projection leads ingroup members to perceive themselves as more prototypical/representative of the superordinate category than the outgroup, a phenomenon defined as higher relative ingroup prototypicality (Mummendey and Wenzel, 1999). Subgroups under a common superordinate category occur naturally in mergers (with the pre-merger group being the ingroup, the merger partner the outgroup and the superordinate category the post-merger group), offering a real setting to study ingroup projection. These projection phenomena have been studied in different groups: students (Wenzel et al., 2003), ethnic groups (Devos and Banaji, 2005; Devos et al., 2010), national groups (Waldzus et al., 2004; Imhoff et al., 2011), self-selected groups like bikers (Waldzus et al., 2004), and even all humankind (Reese et al., 2012).

In the case of organizations, research has shown that compatibility of pre- and post-merger organizations increases ingroup projection (Riketta and Nienaber, 2007), and relative ingroup prototypicality predicts post-merger identification (Gleibs et al., 2008). Although there is often disagreement between lower- and higher-status groups about the relative ingroup prototypicality assigned by/to each other (Waldzus et al., 2004; Devos and Banaji, 2005), ingroup projection is less likely to occur in lower-status groups due to reality constraints implied in the lower-status position (Alexandre et al., 2016a), for instance, less power in negotiations. When disagreement occurs, it is expressed as lower-status groups perceiving their relative ingroup prototypicality not as low as the high-status group perceives (Waldzus et al., 2004; Devos and Banaji, 2005), but rarely to the point of claiming higher relative prototypicality than the majority (Alexandre et al., 2016a). If pre-merger status is relatively low, one can expect that pre-merger prototypicality is also low, which may raise the fear in the lower-status merger partner of being also low in prototypicality in the post-merger group.

Given that lack of commitment to a change process can seriously undermine merger success (Schweiger and Denisi, 1991), one important question is whether it is possible to raise relative ingroup prototypicality for premerger lower-status partners. So far, little is known about contexts allowing for that. Research has shown that minorities’ relative ingroup prototypicality can be increased if the superordinate category is defined as more complex and/or inclusive (Alexandre et al., 2016b) or if the lower-status group is based on strong beliefs that provide a positive social identity regardless of status (Alexandre et al., 2016a). Moreover, mergers are dynamic processes in which the intergroup relations can potentially change. In such dynamic intergroup relations, relative ingroup prototypicality can be claimed by minorities despite their pre-change lower-status position (Alexandre et al., 2016a). Similarly, in an organizational merger, one might think about undermining the heuristic that post-merger prototypicality depends on pre-merger status by introducing specific information that feeds in alternative sources for relative ingroup prototypicality in spite of lower pre-merger status.

### Strategic Fit: Functional Indispensability

Ingroup projection can be perceived not only as relative ingroup prototypicality but also as relative ingroup indispensability (Tseung-Wong and Verkuyten, 2010), “the extent to which particular groups are considered defining parts of the compositional whole, or constitutive for the social identity” (Verkuyten et al., 2014, p. 2). Also, perceptions of lower-status groups’ indispensability are related to the endorsement of more inclusive identities (Mummendey and Wenzel, 1999; Verkuyten et al., 2014; Guerra et al., 2015). Thus, the consequences of ingroup projection such as negative attitudes toward outgroups can be reduced when higher-status groups perceive the lower-status outgroup (e.g., immigrants) as indispensable (Verkuyten et al., 2014; Verkuyten and Martinovic, 2016). Recently, separate dimensions on which groups can be recognized as indispensable have been established: identity indispensability (involving groups’ definitional characteristics) and functional indispensability (involving groups’ contributions) (Guerra et al., 2015, 2016). Identity indispensability mirrors Tseung-Wong and Verkuyten’s (2010) category indispensability and relates to how necessary the ingroup is as a complementary part to define the superordinate category (Guerra et al., 2015). Functional indispensability is based on research on groups’ superordinate goals (Sherif et al., 1961) and on team members’ efforts (Weber and Hertel, 2007). It is defined as the perceived instrumentality of the group’s contribution to a superordinate outcome (Guerra et al., 2015, 2016) (e.g., how much does a social group contribute to the superordinate group’s results).

The relevance of identity indispensability for mergers has been theoretically noted (Verkuyten et al., 2014). One can easily admit immigrants’ important contribution to a national economy, yet still consider them less citizens of that nation. However, when a group’s definition is closely linked to its function based on its goal-directed achievements, as in organizations, functional indispensability can be connected to projected continuity—what the employee can do to achieve future goals (Ullrich et al., 2005; Lupina-Wegener et al., 2013). We assume that this is particularly important for members of pre-merger lower-status partners because usually they do not observe as much continuity between past and present identity as the higher-status counterpart does. When intergroup mergers happen in an organizational context, they are not only dynamic processes with the potential of bringing change in the intergroup relations, but also processes with clear objectives/goals in mind (e.g., financial gains, increase services, get access to a wider client pool, etc.). Thus, we assume that it is not only pre-merger status that matters for relative ingroup prototypicality, but also merger partners’ particular contribution to the merger’s objectives/goals. Whereas pre-merger status offers an *a priori* comparison dimension legitimizing asymmetries and dominance in the merger, functional indispensability provides a future-oriented perspective of contribution to the growth or betterment of the superordinate category, which can be, in itself, a source of positive social identity. More precisely, we propose functional indispensability as a prototypicality booster for lower-status groups: because of the ingroup’s contribution, the superordinate identity will be more positive, leading its members to claim prototypicality within the superordinate category. Indeed, when employees perceive the merger as a growth opportunity, they are more positive about it (Teerikangas, 2010; Paustian-Underdahl et al., 2017). Thus, organizational mergers offer an interesting context to examine group instrumentality toward a superordinate goal as a prototypicality cue.

Groups often merge to grow or complement each other. Complementarity can be seen as synergy or contingency/reciprocity (Gouldner, 1960; Milgrom and Roberts, 1995); or coordination (Fiske, 2000). This framework makes sense for dominant partners, as they often merge to get something complementary from the low-status partner (Jemison and Sitkin, 1986). However, at least at an intragroup level, contribution to an instrumental goal is particularly effectual for the lower-status members (Hertel et al., 2008): low-status but indispensable team members tend to increase their efforts to meet the group goals, compared to when they are not indispensable. We assume that what can be seen by the higher-status partner as complementarity (e.g., enhancing combined effects by getting access to clients or services) can be seen as functional indispensability (e.g., because of intangible capital) by the lower-status partner, legitimizing representativeness/prototypicality claims and making members of this lower-status partner more willing to contribute/commit to the merger. Considering that functional distinctiveness between merger partners is positive for merger integration (van Leeuwen and van Knippenberg, 2003), and complementary backgrounds are related to good performance (van Oudenhoven and de Boer, 1995), promoting functional indispensability among low-status merger partners could benefit merger support. Therefore, instead of approaching indispensability and prototypicality as parallel process as in previous research (e.g., identity indispensability; Tseung-Wong and Verkuyten, 2010), we propose a functional approach to intergroup relations treating indispensability as a precursor of relative ingroup prototypicality. Pre-merger lower-status partners may see themselves more relatively prototypical in the post-merger group the more they consider the ingroup as functionally indispensable to the post-merger group. Moreover, the higher relative ingroup prototypicality, the stronger post-merger identification should be envisioned, and the stronger should be the commitment to merger changes.

### The Role of Information Processing

Positive reactions to a merger announcement, such as change commitment, may depend on the cognitive-motivational resources available and the consequent elaboration of the information. While most research focuses on the integration phase, when merger changes are actually visible, comparatively little is known regarding the pre-merger phase, when the merger is announced (Teerikangas, 2010), other than the fact that it has an impact on how employees support the merger (Buono et al., 1985; Cartwright, 2012). More precisely, when a merger is announced, employees reach a peak of stress and anxiety (Cartwright, 2012); feel anger, rejection, and disappointment (Burlew et al., 1994); get concerned about their situation (Marks and Mirvis, 1985); and seek for information when little is available (Stahl and Sitkin, 2010). This perceived loss of control over their own job might make employees distance themselves from the issue of merger in order to regain control. In turn, this distancing might strike back, as it leads to an increase in stress, lower productivity and work satisfaction, as well as lower post-merger identification (Appelbaum et al., 2000).

This is particularly important when considering that information on merger intentions is often received and processed under circumstances of high cognitive load and time constraints (e.g., Hambrick et al., 2005). Quite frequently, leaders take time and effort to create a vision for the post-merger organization, involving both intellectual and emotional resources, but after announcing the merger to employees, they act as if employees should be embracing that vision in a fraction of that time (Kotter, 2012). For instance, information about the merger is squeezed in routine communication that is not duly processed.

In the framework of dual-process models of information processing, there are two systems operating when individuals are involved in making judgments, decisions, solving problems, and other tasks. One is a quick and effortless mode based on heuristics, and the other is controlled, effortful based on systematic reasoning (Smith and DeCoster, 2000). Within one of such approaches, the heuristic-systematic model of information processing (Chaiken and Trope, 1999), previous research with high-status groups has shown that a group’s status security moderates the effect of information processing on relative ingroup prototypicality: when participants perceived the intergroup relation as secure (stable, legitimate), prototypicality increased when using heuristics. Conversely, when they perceived the intergroup relation as insecure, prototypicality increased under systematic information processing (Rosa and Waldzus, 2012). This is due to group members in insecure intergroup relations being motivated to achieve or maintain high relative ingroup prototypicality, while in secure intergroup relations, they are less directionally motivated in their information processing.

In the context of mergers, information about the merger pattern (whether it will perpetuate status differences or not) has been found to influence relative ingroup prototypicality of the low-status merger partners’ participants, but only when they were under systematic processing (given time to ponder the information and scrutinize prototypicality cues) (Rosa et al., 2017). However, it is unknown whether indispensability information requires systematic (high elaboration) information processing as well, or whether it can simply serve as prototypicality cue impacting relative ingroup prototypicality heuristically (low elaboration) (Chaiken and Trope, 1999). We suspect that the situation of a lower-status merger partner receiving information about own functional indispensability might produce an insecure intergroup relation in the sense that they may process that information motivated by the possible gain in prototypicality.

Consequently, we aim at exploring the role of information processing in employees’ prototypically judgments about the merger, whether the moderation previously found for the effect of merger patterns on relative ingroup prototypicality similarly applies to indispensability information.

### Overview of Present Research

**FIGURE 1:**
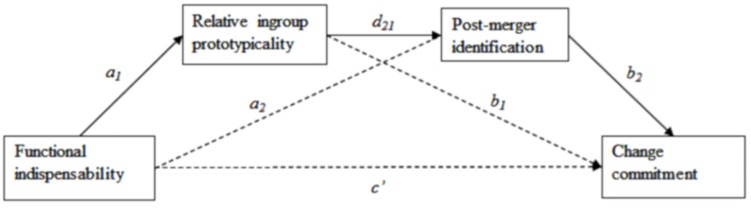
Conceptual model of H2.

In sum, the purpose of this research was to test whether functional indispensability influences lower-status merger partners’ perceptions of being represented/prototypical in the merged group and their support of the merger. We propose that higher functional indispensability will allow lower-status merger partners to claim relative ingroup prototypicality, and, in turn, increase post-merger identification, and merger commitment. We hypothesize that in a merger: (H1) high functional indispensability will increase relative ingroup prototypicality in pre-merger lower-status partners; (H2) relative ingroup prototypicality and post-merger identification will mediate the relation between functional indispensability and change commitment, with higher relative ingroup prototypicality leading to higher identification and then to stronger change commitment (Figure 1). Additionally, we explore whether the relation between indispensability and relative ingroup prototypicality is stronger (i.e., more positive) under systematic vs. heuristic information processing (H3).

These hypotheses were experimentally tested using a real situation of a university consortium potentially aiming to merge (Study 1) and with scenarios describing a merger situation (Studies 2–5). Studies 1–3 focused on the low-status partner, whereas Studies 3–5 also manipulated status, aiming at showing functional indispensability as different from a status element. All procedures followed in all studies were in accordance with the ethical standards of the American Psychological Association and with the Helsinki Declaration of 1975, as revised in 2008. Slight differences in degrees of freedom in analyses from the same study are due to missing data. Overall, ethical approval was granted by the Erasmus Research Institute of Management (Erasmus University Rotterdam), and specific approval for Study 2 was granted from University of Salzburg.

## Study 1

Using the real case of a university consortium potentially leading to a merger, we targeted business students from the lower-status partner university, allowing for a credible manipulation of the university’s functional indispensability, based on real characteristics of both Universities.

### Materials and Methods

#### Participants

Business students from Rotterdam School of Management (Erasmus University Rotterdam) took part in exchange for course credit. Dutch nationality was a stated requirement in the study announcement, to ensure knowledge of different Dutch Universities, but only approximately half of the targets were Dutch. Since all participants were enrolled at the lower-status Dutch university, we included nationality (i.e., Dutch versus non-Dutch) as an additional factor in the analyses. The sample consisted of 479 (263 Dutch) business students, aged 18–29 years (*M* = 19, *SD* = 1.53), 122 reported to be male and 255 female (the remaining did not answer).

#### Design and Procedure

The study followed a 3 (Functional indispensability: high vs. low vs. control) × 2 (Information processing: heuristic vs. systematic) between-participants design. To this design, we added the national background of our participants (Dutch vs. Non-Dutch), resulting in a 3 × 2 × 2 between-participant design for our analyses. Participation was computer-based at the university lab. Information was presented about the alliance between Erasmus University Rotterdam (ingroup) and Leiden University (outgroup). The ingroup was portrayed (consistent with pre-tested popular consensus among Erasmus University Rotterdam students) as the lower-status partner using naturally occurring arguments: it is younger (established in 1913 vs. 1575) and lower-ranked (per the reputation-based Times Higher Education World University Rankings) compared to Leiden University. This information was followed by the experimental manipulations. Participants then completed the dependent measures and were thanked and debriefed upon completion.

#### Manipulating Functional Indispensability

In the high-indispensability condition, participants read that although Leiden University took the lead, only Erasmus University Rotterdam had a business school (Rotterdam School of Management), attracting vast private funding, as well as prestige and for that reason, their contributions were indispensable for the overall Consortium University outcomes. Conversely, in the low-indispensability condition, participants read that Leiden University takes the lead, since its contributions to the consortium were massive regarding prestige and funding in natural sciences, as well as law and politics. These areas are absent or less covered by Erasmus University Rotterdam, giving rooms to state their contributions as weaker and, therefore, not indispensable for the overall Consortium University outcomes. No indispensability information appeared in the control condition.

#### Manipulating Information Processing

Before dependent measures, instructions were given to take time and ponder while answering (no time pressure/systematic condition) or that to mimic everyday life, time to answer was constrained (time pressure/heuristic condition), by a countdown chronometer displayed for each question (participants could still answer after countdown was over).

#### Measures

Unless otherwise stated, measures used a seven-point scale from “1 = Strongly disagree” to “7 = Strongly agree.” Change commitment was asked as anticipated commitment.

#### Relative Ingroup Prototypicality

A pictorial measure was used, based on “inclusion of other in the self” scales (e.g., Aron et al., 1992) adapted to the group level (Schubert and Otten, 2002) and then adapted to comparisons within a superordinate category (Waldzus and Mummendey, 2004). It is a set of two figures, each with seven pairs of circles decreasing in distance, from 1 (“Not at all representative”) to 7 (“Strongly representative”). One figure was presented for ingroup—superordinate category, and the other for outgroup—superordinate category. A second pictorial measure was included, depicting relative ingroup prototypicality as centrality. Two figures (for ingroup and outgroup) were shown, each containing seven concentric circles, with the central circle representing the superordinate category (Rosa and Waldzus, 2012). For both measures, relative ingroup prototypicality corresponds to the difference between ingroup and outgroup scores. The two measures were strongly correlated, *r*(477) = 0.56, *p* < 0.001. Thus, we created a composite score with the mean of both measures.

#### Post-merger (Superordinate) Identification

A six-item organizational identification scale (Mael and Ashforth, 1992) was used (α = 0.78; e.g., “*When someone criticizes the Consortium University, it feels like a personal insult”*).

#### Perceived Relative Status

A pictorial measure was used (Rosa and Waldzus, 2012), with two vertical arrows divided into seven portions, representing ingroup and outgroup’s status (the lowest portion representing very low status and the highest portion representing very high status).

#### Change Commitment

The scale from Fedor et al. (2006) assessing intentions to act on behalf of the change was used (α = 0.90; e.g., “*I will be fully supportive of this change*”).

#### Additional Measure

A single item asked “*How much do you agree with the consortium between Erasmus University Rotterdam and Leiden University*?”^2^

#### Manipulation Checks

An item from Rosa and Waldzus (2012) was used to check the information processing manipulation (e.g., “*While answering these questions so far, I have been:*…*under time pressure*”). The indispensability manipulation was checked via the following item: “*In the beginning, when you read information about the terms of the consortium between Erasmus University Rotterdam and Leiden University*—*Which university contributes most to the consortium university?*”

### Results and Discussion

#### Preliminary Analyses

Participants perceived more time pressure in the heuristic condition (*M* = 4.00, *SD* = 1.80) than in the systematic condition (*M* = 2.21, *SD* = 1.32). A 3 × 2 × 2 GLM with both manipulations and nationality as factors and the information processing manipulation check as dependent variable showed just a main effect of information processing, *F*(1,465) = 146.65, *p* < 0.001, η^2^*_*p*_* = 0.24.

There was a significant effect of indispensability condition on the manipulation check, *F*(2,465) = 10.44, *p* < *0.001*, η^2^*_*p*_* = 0.04. Simple paired comparisons showed that participants perceived their university as contributing significantly more to the consortium university in both the high indispensability condition (*M* = 3.50, *SD* = 1.41) and the control condition (*M* = 3.36, *SD* = 1.22) than in the low indispensability condition (*M* = 2.82, *SD* = 1.40), *p*’s < 0.001. However, the control and the high indispensability conditions were not different (*p* = 0.45). Also, indispensability interacted with nationality, *F*(2,465) = 3.92, *p* = 0.02, η^2^*_*p*_* = 0.02: for Dutch participants, high indispensability was different from low and control (*p*’s < 0.01), but not for non-Dutch (*p*’s > 0.07). The manipulation check item was probably too reductionist, because it asked just which university had most contribution, without connection to the contribution’s perceived instrumentality.

Finally, participants perceived their ingroup as lower in status compared to the outgroup (*M* = −0.67, *SD* = 1.27), regardless of experimental condition (*p*’s > 0.11), but the non-Dutch perceived lower status (*M* = −0.79, *SD* = 1.43) than the Dutch (*M* = −0.57, *SD* = 1.11), *F*(1,465) = 4.14, *p* = 0.04, η^2^*_*p*_* = 0.01.

#### Hypotheses’ Testing

To test whether high functional indispensability affected prototypicality perceptions (H1), and exploring whether and how this effect was moderated by information processing (H3), a 3 (Functional indispensability) × 2 (Information processing) × 2 (Nationality) GLM was run with relative ingroup prototypicality as the dependent variable. Results showed a main effect of indispensability *F*(2,465) = 5.81, *p* = 0.003, η^2^*_*p*_* = 0.02. Simple paired comparisons showed a significant difference in the high vs. low indispensability conditions and the control vs. low (*p*’s < 0.02) but not high vs. control (*p* = 0.40). It was not qualified by an interaction with information processing, *F*(2,465) = 0.34, *p* = 0.71, η^2^*_*p*_* = 0.001 (Table 1).

**TABLE 1:** Means and standard deviations of relative ingroup prototypicality, depending on functional indispensability (Studies 1–5), information processing (Studies 1–3 and 5), and status (Studies 3–5).

				**Study 1**			**Study 2**					**Study 3**			**Study 4**			**Study 5**		
				***M***	***SD***	***N***	***M***	***SD***	***N***			***M***	***SD***	***N***	***M***	***SD***	***N***	***M***	***SD***	***N***
Indispensability	Low/No	Information processing	Systematic	−0.67	0.89	80	−1.85	1.99	26	Status	Low	−0.74	1.84	34	−0.25	1.63	29	−0.60	1.83	31
											High	0.64	1.69	33	1.07	1.19	29	0.95	1.68	28
			Heuristic	−0.54	1.17	81	−0.79	2.15	42		Low	−0.77	1.60	29				−0.66	1.94	29
											High	0.58	1.71	30				0.67	1.70	34
	Control		Systematic	−0.27	0.94	82	–	–	–		Low	–	–	–	–	–	–	–	–	–
											High	–	–	–	–	–	–	–	–	–
			Heuristic	−0.15	1.19	75	–	–	–		Low	–	–	–	–	–	–	–	–	–
											High	–	–	–	–	–	–	–	–	–
	High		Systematic	−0.30	1.06	76	−0.74	2.12	39		Low	−0.35	1.39	28				1.40	1.24	27
											High	1.22	1.75	30				1.65	1.36	26
			Heuristic	−0.32	1.08	83	−1.60	2.03	43		Low	−0.08	1.67	36	1.19	1.51	25	1.52	1.74	27
											High	1.08	2.05	32	1.18	1.79	30	1.86	1.21	27

There was a main effect of nationality, *F*(2,465) = 4.50, *p* = 0.03, η^2^*_*p*_* = 0.01, with lower relative ingroup prototypicality for Dutch (*M* = −0.48, *SD* = 0.93) vs. non-Dutch (*M* = −0.25, *SD* = 1.21) participants, but no interaction of nationality with other factors.

Finally, testing whether relative ingroup prototypicality and post-merger identification mediate the relation between functional indispensability and commitment to change (H2), we estimated the predicted indirect effect using the PROCESS SPSS Macro created and documented by Hayes (2017). Functional indispensability had three levels, and we used Helmert coding to create two orthogonal contrasts representing the variable in the model, given the results of the manipulation check. We requested 10,000 bootstrap samples to estimate indirect effects’ confidence intervals (seed = 21092109). Because prototypicality had a different scale compared to identification and commitment, variables were standardized prior to the analysis. Because functional indispensability is not continuous, partially standardized indirect effects were also requested (see Hayes, 2017, for further explanations about these procedures). Information processing and nationality were controlled as covariates.

Results (Table 2) show that functional indispensability predicted relative ingroup prototypicality, which marginally predicted post-merger identification (*p* = 0.06), which predicted change commitment partially as hypothesized (H2). Moreover, the predicted indirect effect was marginally significant, β = 0.01, *CI* [−0.002,0.03]. All these effects refer to low vs. control and high indispensability, but not control vs. high indispensability, for which all effects were non-significant (Table 2).

**TABLE 2:** Summary of sequential mediation, Study 1 (Figure 1 for paths).

**Outcome**												
		**Relative ingroup prototypicality (M1)**				**Post-merger identification (M2)**				**Change commitment (Y)**		
**Antecedent**		**Coeff.**	***SE***	***p***		**Coeff.**	***SE***	***p***		**Coeff.**	***SE***	***p***
Functional indispensability (X1) (low vs. control and high)	*a*_1 (x1)_	0.31	0.10	0.001	*a*_2 (x1)_	–0.02	0.10	0.83	*c′_(x1)_*	–0.07	0.09	0.39
Functional indispensability (X2) (control vs. high)	*a*_1 (x2)_	–0.08	0.11	0.45	*a*_2 (x2)_	–0.13	0.11	0.24	*c′_(x2)_*	–0.04	0.10	0.66
Relative ingroup prototypicality		–	–	–	*d*_21_	0.09	0.05	0.06	*b*_1_	–0.02	0.04	0.64
Identification		–	–	–		–	–	–	*b*_2_	0.49	0.04	<0.001
Constant		–0.29	0.14	0.04		–0.49	0.14	0.001		0.01	0.13	0.93
Nationality		0.20	0.09	0.03		0.34	0.09	0.0003		–0.01	0.08	0.93
Processing		0.03	0.05	0.47		0.01	0.05	0.82		–0.01	0.04	0.90
		*R*^2^ = 0.04				*R*^2^ = 0.04				*R*^2^ = 0.24		
		*F*(4,472) = 4.36, *p* = 0.002				*F*(5,471) = 4.10, *p* = 0.001				*F*(6,470) = 24.83, *p* < 0.001		

**Partially standardized relative indirect effects**		**Coeff.**				***Boot SE***		***Lower level Boot CI***		***Upper level Boot CI***		

*a_1_ b_1_*	*x1*	−0.01				0.02		−0.05		0.02		
	*x2*	0.002				0.01		−0.01		0.02		
*a_2_ b_2_*	*x1*	−0.01				0.05		−0.10		0.08		
	*x2*	−0.06				0.05		−0.17		0.04		
*a_1_ d_21_ b_2_*	*x1*	0.01				0.01		−0.002		0.03		
	*x2*	−0.004				0.01		−0.02		0.01		

Additional GLMs of the indispensability on post-merger identification and on change commitment were conducted. There was no main effect of functional indispensability on post-merger identification *F*(2,471) = 0.70, *p* = 0.50, η^2^*_*p*_* = 0.003, there was just a main effect of nationality *F*(2,471) = 14.96, *p* < 0.001, η^2^*_*p*_* = 0.03, with lower identification for Dutch (*M* = 3.82, *SD* = 1.07) vs. non-Dutch (*M* = 4.20, *SD* = 1.01). There were no effects of indispensability on change commitment, *F*(2,471) = 0.74, *p* = 0.48, η^2^*_*p*_* = 0.003.

In sum, these results showed preliminary evidence of the impact of functional indispensability on relative ingroup prototypicality (H1). Increased relative ingroup prototypicality was marginally related to post-merger identification and then to change commitment (H2). However, the effect of indispensability seemed not to be influenced by information processing modes (H3). A possible explanation may be found in both the sample and the context. Although university students tend to identify with their university (e.g., Gleibs et al., 2013), they might have seen just the bright side of the consortium, as they agreed with it (*M* = 4.86, *SD* = 1.42) similarly across experimental conditions (*p*’s > 0.12). Nationality (implying awareness of Dutch universities) also impacted results. More precisely, we tested the hypotheses for Dutch and non-Dutch separately, and results are similar, yet stronger, for the Dutch sample, but are not significant (not supporting any of the hypotheses) for the non-Dutch sample. In a way, this was our *a priori* assumption as we aimed to collect responses only from Dutch students. Also, the effect of indispensability on relative ingroup prototypicality was found only partially due to problems with the indispensability manipulation (high = control condition). More importantly, the information provided about status and indispensability was problematic, because we relied on context-specific, naturally occurring information. Status was always low, but presented as prestige (age and reputation rankings), which is disconnected from the notion that status differences in mergers derive mostly from economic power factors (Giessner et al., 2006). More problematic was the fact that naturally occurring arguments for indispensability also included status-related information (prestige). Although it did not influence actual status perceptions, it is an important confound to be addressed. Thus, the subsequent studies tested our hypotheses with a merger scenario between two fictitious companies.

## Study 2

Although using a real-life situation, Study 1 surveyed a student population about an ongoing alliance (not merger) between two universities. We suspect that the positivity of the situation for students made this a less-than-ideal test of our hypotheses. Study 2 surveyed employees online, using a merger scenario. Based on the critical incident technique (Flanagan, 1954), we simulated a merger announcement, aiming at keeping everything constant except the experimental manipulations. This is a standard methodological choice to test causality and grant internal validity, and has been widely used in merger research, especially when the focus is—like in our case—on expected reactions (e.g., Giessner et al., 2006; Thorbjørnsen and Dahlén, 2011).

Also, instead of low, control (no information), and high levels of functional indispensability, the design was simplified by focusing on only two levels of functional indispensability (present or absent). Status was unambiguously presented as low.

### Materials and Methods

#### Participants

Of 290 people who were recruited via professional social networks and started the survey, 150 completed it. Twelve were excluded upon reporting being students. After conducting missing data analyses and verifying that results do not change with multiple imputations, data from participants who did not respond the dependent measures were removed. The final sample consisted of 150 workers, ages 22–66 (*M* = 44; *SD* = 9.95); 59 reported being male and 77 female (the remaining did not answer). Data were collected in Croatia and Austria/Germany (83 and 67 participants, respectively), with measures being translated from English into the respective native languages by native speakers, followed by back-translation. Country was included as factor in the analyses.

#### Design and Procedure

The study followed a 2 (Indispensability: no indispensability vs. indispensability) × 2 (Information processing: Heuristic vs. systematic) between-participants design. Data were collected online (Qualtrics, Provo, UT, United States). Participants were thanked and debriefed online upon study completion. The intergroup situation was created via a written scenario, adapted from Giessner et al. (2006). Based on a critical incident technique (Flanagan, 1954), participants were asked to imagine they work at a company that was going to merge with another company. Status information was constant, depicting the ingroup’s company (BOLT) as lower than the outgroup (ACME) on several status-related indicators: stock-market share, transaction volume, and annual profit. To provide realism, the scenario concluded with a statement about the possibility of layoffs or demotions because of the merger. Therefore, the setting aimed at being not as positive as in Study 1.

#### Experimental Manipulations

The scenario included indispensability information, right after the subgroup comparison. In the indispensability condition, participants learned that BOLT holds the patent of a new technology and ACME intends to integrate BOLT’s expertise to provide better services for customers, together with fictitious data depicting BOLT’s high technological market-share. It would be unrealistic for a fictitious memorandum to have a clear low indispensability condition, which could produce threat perceptions more than low indispensability. Instead, we created a no-indispensability condition, with no information about a patent shown, and fictitious data depicting BOLT as having similar technological market-share to ACME.

Information processing was manipulated as in Study 1.

#### Measures

Measures were the same as in Study 1 (α = 0.90 for change commitment), with five exceptions. First, as the items from Mael and Ashforth (1992) require knowledge about the group, a three-item measure based on Giessner (2011) was used to measure identification (e.g., I would identify myself with the merged organization, α = 0.78). Second, since participants had no prior knowledge about the information in the scenario, questions were asked in terms of what participants expected to be their identification, prototypicality, commitment, etc. Third, for the functional indispensability manipulation check, we used four items adapted from the Functional Indispensability Scale (Guerra et al., 2016) (α = 0.64). Fourth, only one pictorial measure of relative ingroup prototypicality was presented. Fifth, other check measures such as years of employment and education level, as well as some additional measures (such as security, pre-merger ingroup identification and the same attempted verbal scale as in Study 1) were included in the questionnaire but not analyzed.

Data were analyzed with participants’ own experience of merger and no effects (main or interaction) were found in the analyses (*p*’s > 0.37). Therefore, the variable was not included in the final analyses.

### Results and Discussion

#### Preliminary Analyses

A 2 × 2 × 2 GLM with both manipulations and country as factors, and the information processing manipulation check as the dependent variable showed just a main effect of time pressure, *F*(1,145) = 45.47, *p* < 0.001, η^2^*_*p*_* = 0.24: more time pressure in the heuristic condition (*M* = 4.41, *SD* = 1.75) than in the systematic condition (*M* = 2.62, *SD* = 1.53). A 2 × 2 × 2 GLM with both manipulations and country as factors and the indispensability manipulation check as the dependent variable showed just a main effect of indispensability, *F*(1,148) = 5.44, *p* = 0.02, η^2^*_*p*_* = 0.04: functional indispensability was higher in the indispensability condition (*M* = 5.15, *SD* = 0.82) than in the no-indispensability condition (*M* = 4.89, *SD* = 0.82).

Finally, participants perceived their ingroup status to be lower compared to the outgroup [*M* = −1.07, *SD* = 1.60; *t*(151) = −8.27, *p* < *0.001*], regardless of experimental conditions (*p*’s > 0.15).

#### Hypotheses’ Testing

**FIGURE 2:**
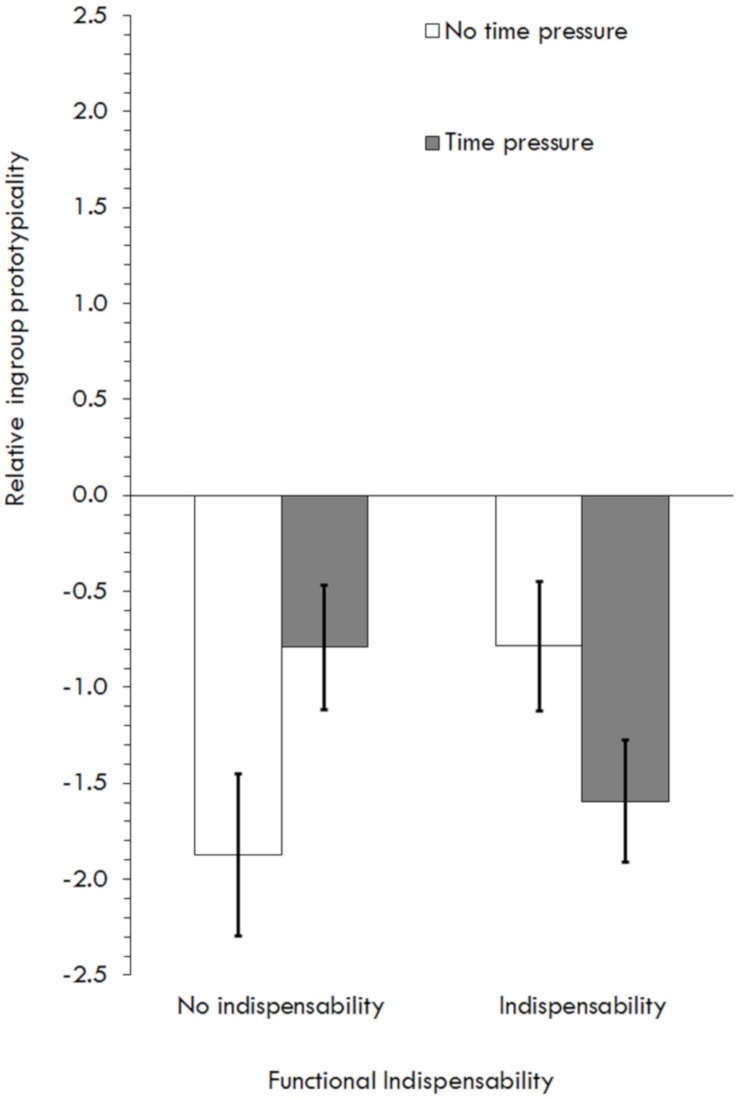
Effects of relative ingroup prototypicality depending on functional indispensability and information processing in Study 2.

Testing H1 and exploring H3, a 2 (Functional indispensability) × 2 (Information processing) GLM showed no main effect of functional indispensability on prototypically perceptions, *F*(1,142) = 0.17, *p* = 0.69, η^2^*_*p*_* = 0.001. Instead, an interaction with information processing (H3) was found, *F*(1,142) = 7.19, *p* = 0.01, η^2^*_*p*_* = 0.05 (Figure 2). Simple main effects showed an effect of time pressure in the no-indispensability condition, *F*(1,142) = 4.15, *p* = 0.04, η^2^*_*p*_* = 0.03, and not in the indispensability condition, *F*(1,142) = 3.04, *p* = 0.08, η^2^*_*p*_* = 0.02. More interesting for our predictions, simple main effects also showed no effect of indispensability in the heuristic condition, *F*(1,142) = 3.11, *p* = 0.08, η^2^*_*p*_* = 0.02, and a significant and positive effect in the systematic condition, *F*(1,142) = 4.08, *p* = 0.045, η^2^*_*p*_* = 0.03, suggesting that the effect of indispensability on relative ingroup prototypicality in this study was contingent on systematic rather than heuristic information processing (Table 1).

Finally (H2) was tested as in Study 1 but in a more integrated way: a sequential mediation moderated by information processing in the first path (Model 83 by Hayes, 2017, Table 3; variables were standardized or mean centered and 10,000 bootstrap samples were requested to estimate indirect effects, seed = 21092019). Consistent with H2, when the interaction was added, there was a statistically significant increase in explained variance (Table 3). The effect of indispensability on prototypicality was significant under systematic processing, β = 0.27, *p* = 0.03, *CI*_95_ [0.02,0.51], and not under heuristic processing, β = −0.18, *p* = 0.09, *CI*_95_ [−0.39,0.03]. The indirect effect of indispensability on change commitment via relative ingroup prototypicality and post-merger identification was significant in the systematic condition, β = 0.02, *CI*_95_ [0.001,0.04], but not in the heuristic condition, β = −0.01, *CI*_95_ [−0.04,0.001]. The index of moderated mediation was not significant, β = −0.03, *CI*_95_ [−0.07,−0.003] (Table 3).

**TABLE 3:** Summary of sequential mediation (H2) moderated by information processing, Study 2.

**Outcome**												
		**Relative ingroup prototypicality (M1)**				**Post-merger identification (M2)**				**Change commitment (Y)**		
**Antecedent**		**Coef.**	***SE***	***p***		**Coef.**	***SE***	***p***		**Coef.**	***SE***	***p***
Functional indispensability (X)	*a*_1_	0.01	0.08	0.89	*a*_2_	–0.09	0.08	0.26	*c′*	–0.06	0.08	0.40
Information processing (W)		0.01	0.08	0.91		–	–	–		–	–	–
Relative ingroup prototypicality		–	–	–	*d*_21_	0.24	0.08	0.003	*b*_1_	0.19	0.08	0.01
Identification		–	–	–		–	–	–	*b*_2_	0.25	0.08	0.002
Constant		–0.32	0.25	0.19		0.73	0.24	0.003		–0.89	0.24	<0.001
Country		–0.24	0.16	0.14		–0.48	0.16	0.002		0.62	0.16	<0.001
Indispensability × Processing		–0.23	0.08	0.01		–	–	–		–	–	–
		*R*^2^ = 0.06				*R*^2^ = 0.14				*R*^2^ = 0.17		
		*F*(4,145) = 2.47, *p* = 0.05				*F*(3,146) = 7.81, *p* < 0.001				*F*(4,145) = 7.60, *p* < 0.001		
Change		*R*^2^ = 0.05										
		*F*(1,145) = 7.60, *p* = 0.01										

**Conditional indirect effects**		**Coef.**				***Boot SE***		***Lower level Boot CI***		***Upper level Boot CI***		

	*a_2_ b_2_*	−0.02				0.02		−0.07		0.02		
At systematic processing	*a_1_ b_1_*	0.05				0.04		−0.001		0.15		
	*a_1_ d_21_ b_2_*	0.02				0.01		0.001		0.04		
At heuristic processing	*a_1_ b_1_*	−0.04				0.03		−0.10		0.01		
	*a_1_ d_21_ b_2_*	−0.01				0.01		−0.04		0.001		
Index of moderated mediation	*a_1_ b_1_*	−0.09				0.06		−0.21		0.003		
	*a_1_ d_21_ b_2_*	−0.03				0.02		−0.07		−0.003		

Additional GLMs of indispensability on post-merger identification and on change commitment were conducted. Functional indispensability had no effect on post-merger identification, *F*(1,148) = 1.57, *p* = 0.21, η^2^*_*p*_* = 0.01; however, there was a significant interaction between indispensability, processing, and country sample. Simple main effects showed no significant effects of indispensability on post-merger identification at any levels of the other variables (*p*’s > 0.09) but showed (1) simple effects of information processing on post-merger identification at high indispensability for both the Croatian sample (*p* = 0.03) and the German/Austrian sample (*p* = 0.04), (2) simple effects of country sample at low indispensability systematic conditions (*p* = 0.01), low indispensability heuristic conditions (*p* = 0.04), and high indispensability heuristic conditions (*p* > 0.001). Moreover, functional indispensability had no effect on change commitment, *F*(1,148) = 0.73, *p* = 0.40, η^2^*_*p*_* = 0.01.

In sum, results support that functional indispensability can account for higher relative ingroup prototypicality when given enough time to process the information (H3), and, accordingly, the expected link between indispensability and change commitment via relative ingroup prototypicality and post-merger identification (H2) was supported under systematic processing. This corroborates previous findings pointing to the need of providing employees with enough time/resources to process merger announcement information (Rosa et al., 2017). There was no main effect of indispensability on relative ingroup prototypicality (H1). Although this study successfully manipulated indispensability with less status-related information, information concerning indispensability was minimal (one paragraph) compared with status information (one page) and visually different. This could have impacted the results, and Study 3 aimed at overcoming these limitations.

## Study 3

To disentangle the roles of status and indispensability, Study 3 used the same scenario but included a status manipulation. Therefore, the test of H1 (high functional indispensability having a positive effect on relative ingroup prototypicality for pre-merger lower-status partners) involves not only a main effect of functional indispensability, but also an interaction with status. The indispensability manipulation was improved to better capture the theoretical construal.

### Materials and Methods

#### Participants

Three hundred and thirty-two individuals living in the United Kingdom (UK) participated, but due to a coding error, 66 of them did not see the experimental manipulations (they just saw a block corresponding to the manipulation checks). The final sample consisted of 266 individuals, ages 18–69 (*M* = 46, *SD* = 12.53); 131 reported being male and 135 female.

#### Design and Procedure

The study followed a 2 (Indispensability: no indispensability vs. indispensability) × 2 (Status: High vs. low) × 2 (Information processing: Heuristic vs. systematic) between-participants design. Data were collected through an online survey (Qualtrics, Provo, UT, United States) and distributed via a panel service (Toluna). Participants were thanked and debriefed online and paid after completing the study.

#### Experimental Manipulations

Status was manipulated based on Giessner et al. (2006) scenarios, but to show an unambiguous lower hierarchical standing, participants received graphical information comparing the ingroup (BOLT) with the outgroup (ACME) favorably vs. unfavorably on Sachdev and Bourhis’ (1985) dimensions: size (number of employees), prestige (stock market), and economic power (resources, via capital).

The indispensability manipulation followed, with textual information stating that the contribution of BOLT was indispensable for the outcomes of the merged organization because of technology created and held by BOLT (indispensability) or that BOLT was not indispensable because there were several other companies in the UK with a similar profile.

#### Measures

Relevant measures were the same as in Study 2 (α = 0.88 for change commitment; α = 0.66 for post-merger identification), with some exceptions. First, due to a coding error, the block with the manipulation check intended for status and indispensability was not seen by participants. However, we could still use influence as an alternative measure. Thus, a manipulation check of status, with a single item asking about the influence of BOLT in the merger, compared to ACME (1 = no influence to 7 = high influence). Second, there was a check question of scenario realism (1–7 scale). Third, the relative ingroup prototypicality measure was a composite of the pictorial measure used in Studies 1–2 plus two textual items about the ingroup and two about the outgroup (e.g., *The group I consider to be more representative of the merged company is ACME*). For both measures, relative ingroup prototypicality corresponds to the difference between ingroup and outgroup scores. The two measures were correlated, *r*(232) = 0.38, *p* < 0.001. Thus, we created a composite score with the mean of both measures.

Again, data were analyzed with participants’ own experience of merger as factor and no effects were found (*p*’s > 0.54).

### Results and Discussion

A 2 × 2 × 2 GLM with all manipulations as factors and the status manipulation check as the dependent variable showed just a main effect of status, *F*(1,227) = 21.08, *p* < 0.001, η^2^*_*p*_* = 0.09: status was higher in the high-status (*M* = 3.68, *SD* = 0.96) than in the low-status condition (*M* = 3.02, *SD* = 1.19). No main effect of or interaction with indispensability was found, *p*’s > 0.52. Another GLM with time pressure manipulation check as dependent variable showed just a main effect of time pressure *F*(1,239) = 85.72, *p* < 0.001, η^2^*_*p*_* = 0.26: time pressure was higher in the time pressure condition (*M* = 4.18, *SD* = 1.41) than in the no-time-pressure condition (*M* = 2.59, *SD* = 1.24). No other main effects or interactions were found, *p*’s > 0.22. However, participants in the high-status condition considered the scenario less realistic (*M* = 4.71, *SD* = 1.69) than in the low-status condition (*M* = 5.19, *SD* = 1.42), *F*(1,239) = 6.00, *p* = 0.02, η^2^*_*p*_* = 0.02.

Testing H1 and exploring H3, a 2 (Functional indispensability) × 2 (Status) × 2 (Information processing) GLM on relative ingroup prototypicality showed a main effect of indispensability, *F*(1,244) = 6.12, *p* = 0.01, η^2^*_*p*_* = 0.02, and of status, *F*(1,244) = 38.91, *p* < 0.001, η^2^*_*p*_* = 0.14, on relative ingroup prototypicality. No effects of information processing, or any interaction of any variables, were found, *p*’s > 0.62 (Table 1).

Finally, H2 was tested in a more integrated way: a sequential mediation moderated by status. With regard to which path(s) would be moderated, we reasoned that the role of indispensability and prototypicality might be different for higher- and lower-status groups, but not identification. For that reason, we tested a model representing the moderation of status in all paths except on the relation between identification and commitment [see Hayes (2017) for model customization] (Table 4). Variables were standardized or mean centered and 10,000 bootstrap samples were requested (seed = 21092109) to estimate indirect effects. Information processing was included as covariate. Consistent with results found testing H1, there were just main effects but no interaction between status and indispensability on prototypicality. Regarding effects on post-merger identification, there was an interaction between status and prototypicality (see Table 4). The effect was significant at low status, β = 0.25, *p* = 0.01, *CI* [0.05,0.45], but not at high status, β = −0.09, *p* = 0.33, *CI* [−0.27,0.09]. Regarding effects on commitment, there was just a main effect of identification. The direct effect of prototypicality (i.e., bypassing identification) was not significant and not moderated by status. The predicted indirect effect of indispensability on commitment via prototypicality and identification was not significant at low status, but it approached significance closely, β = 0.02, *CI* [−0.002,0.06], while it was far from being significant, and not even in the same direction, at high-status (Table 4). The index of moderated mediation was not significant, β = −0.03, *CI* [−0.08,0.01].

**TABLE 4:** Summary of sequential mediation, Study 3 (moderated by status).

**Outcome**												
		**Relative ingroup**				**Post-merger**				**Change**		
		**prototypicality (M1)**				**identification (M2)**				**commitment (Y)**		
**Antecedent**		**Coef.**	***SE***	***p***		**Coef.**	***SE***	***p***		**Coef.**	***SE***	***p***
Functional indispensability (X)	*a*_1_	0.15	0.06	0.01	*a*_2_	–0.02	0.06	0.79	*c′*	0.002	0.05	0.97
Status (W)		0.37	0.06	<0.001		–0.03	0.07	0.61		0.01	0.05	0.80
Prototypicality		–	–	–	*d*_21_	0.08	0.07	0.24	*b*_1_	0.05	0.05	0.35
Indispensability × Status		–0.01	0.06	0.93		–0.02	0.06	0.71		–0.02	0.05	0.15
Prototypicality × Status		–	–	–		–0.17	0.07	0.01		0.08	0.06	0.15
Identification		–	–	–		–	–	–	*b*_2_	0.61	0.05	<0.001
Constant		–0.0001	0.60	0.99		0.06	0.07	0.36		–0.04	0.05	0.49
Information Processing		–0.002	0.60	0.99		0.13	0.06	0.03		0.04	0.05	0.49
		*R*^2^ = 0.16				*R*^2^ = 0.05				*R*^2^ = 0.38		
		*F*(4,246) = 11.29, *p* < 0.001				*F*(6,244) = 1.99, *p* = 0.07				*F*(7,243) = 21.71, *p* < 0.001		
Change		*R*^2^ = 0.00			X × W	*R*^2^ = 0.001			X × W	*R*^2^ = 0.001		
		*F*(1,246) = 0.01, *p* = 0.93				*F*(1,244) = 0.14, *p* = 0.71				*F*(1,243) = 0.20, *p* = 0.65		
		M1 × W				*R*^2^ = 0.02			M1 × W	*R*^2^ = 0.01		
						*F*(1,244) = 6.19, *p* = 0.01				*F*(1,243) = 2.11, *p* = 0.15		

**Conditional indirect effects**		**Coef.**				***Boot SE***		***Lower level 95% Boot CI***		***Upper level 95% Boot CI***		

At low status	*a_1_ b_1_*	–0.005				0.01		–0.03		0.02		
	*a_2_ b_2_*	0.004				0.05		–0.10		0.12		
	*a_1_ d_21_ b_2_*	0.02				0.02		–0.002		0.06		
At high status	*a_1_ b_1_*	0.02				0.02		–0.003		0.07		
	*a_2_ b_2_*	–0.02				0.06		–0.14		0.09		
	*a_1_ d_21_ b_2_*	–0.01				0.01		–0.04		0.02		
Index of moderated mediation	*a_1_ b_1_*	0.02				0.02		–0.01		0.08		
	*a_2_ b_2_*	–0.03				0.08		–0.19		0.12		
	*a_1_ d_21_ b_2_*	–0.03				0.02		–0.08		0.005		

Additional GLMs of indispensability on post-merger identification and on change commitment were conducted. Functional indispensability had no effect on post-merger identification *F*(1,249) = 0.05, *p* = 0.82, η^2^*_*p*_* = 0.001, or change commitment, *F*(1, 250) = 0.03, *p* = 0.86, η^2^*_*p*_* = 0.001. Given that previous theory links status asymmetry to group identification via prototypicality, this particular mediation was also tested (Model 4, Hayes, 2017). Information processing and indispensability were included as covariates. In sum, status positively predicted prototypicality, β = 0.37, *p* < 0.001, but prototypicality alone did not predict identification, β = 0.07, *p* = 0.34, and accordingly, the indirect effect was not significant, β = 0.02, *CI* [−0.04,0.09].

Taking into account the hypotheses’ testing, there was partial support for H1 and H2, and no support for H3. Study 3 offered a replication of the effect of functional indispensability on relative ingroup protoypicality, but the other expected effects were in the predicted direction but not very strong. Importantly, the study presented some problems: (1) scenario realism ratings were significantly lower in the high-status condition and (2) status was manipulated with more information displayed than indispensability was. The goal was to depict BOLT as unambiguously lower than ACME on many dimensions related with status in the literature, but it might have weakened attention paid to the indispensability information. Study 4 attempted to address these problems by fine-tuning the experimental scenarios. The design was simplified by excluding information processing.

## Study 4

### Pre-study

To provide evidence for indispensability as differentiated from status, a pre-study was conducted online (Qualtrics, Provo, UT, United States). The aim was twofold: find the best dimension to manipulate status (therefore reducing the amount of information presented to participants) and test an indispensability manipulation realistic at all levels of status. A total of 132 participants (55 male, 77 female, aged 18–78 years old) (*M* = 40, *SD* = 13.64) read a merger scenario similar to previous studies, mentioning a merger-partner market analysis report, containing status (size or prestige or economic power) and indispensability (indispensable or not) information. Size compared number of employees, prestige compared “best-place-to-work” ranking, and power compared economic resources (capital), each with a comparative graphic. Based on Guerra and colleagues’ (Guerra et al., 2015) definition and business expert advice, the indispensability manipulation included a graphic comparing the growth potential of a merger of ACME with BOLT with the growth potential of a merger of ACME with a third potential partner. It was followed by a concluding remark that BOLT’s contributions were (or were not) indispensable for the merged organization because of a technological patent (indispensability), or because other companies had similar profile/contributions (no indispensability). After reading the scenario, participants were asked about relative status and indispensability as previously, thanked, and debriefed.

Results showed that relative status was affected only by the status manipulation, *F*(1,95) = 39.02, *p* < 0.001, η^2^*_*p*_* = 0.29 (*M* = −1.04, *SD* = 2.41 low; *M* = 1.72, *SD* = 2.06 high) and perceived indispensability was affected only by the indispensability manipulation, *F*(1,95) = 5.06, *p* = 0.03, η^2^*_*p*_* = 0.05 (*M* = 3.85, *SD* = 2.07 low; *M* = 4.75, *SD* = 2.09 high). Moreover, there were no differences on the manipulation check between ways of manipulating status [all forms of manipulating status were equally successful (*p* = 0.14)]. Still, economic power operationalized as resources (capital) produced the biggest asymmetry between high and low perceived relative status (*M* = −1.16, *SD* = 2.22 low; *M* = 2.33, *SD* = 1.67 high), corroborating previous findings that economic power is a meaningful status cue in mergers (Giessner et al., 2006).

### Materials and Methods

Participants were recruited by a panel service (Prolific Academic) and participated online (Qualtrics, Provo, UT, United States). Among a total of 119 participants, 6 stated participation in Study 3 and were, therefore, excluded from analyses, resulting in a final sample of 113 (26 male, 87 female, 21–58 years old) (*M* = 34, *SD* = 8.73). The design was simplified by excluding information processing, resulting in a 2 (Status: High vs. low) × 2 (Indispensability: no indispensability vs. indispensability) between-participants design. Participants read scenario information with the experimental manipulations previously validated in the pre-study (choosing economic power as the basis for status manipulation) and answered the dependent measures, upon which they were thanked, debriefed, and paid. Measures were the same as in Study 3.^3^

### Results and Discussion

**FIGURE 3:**
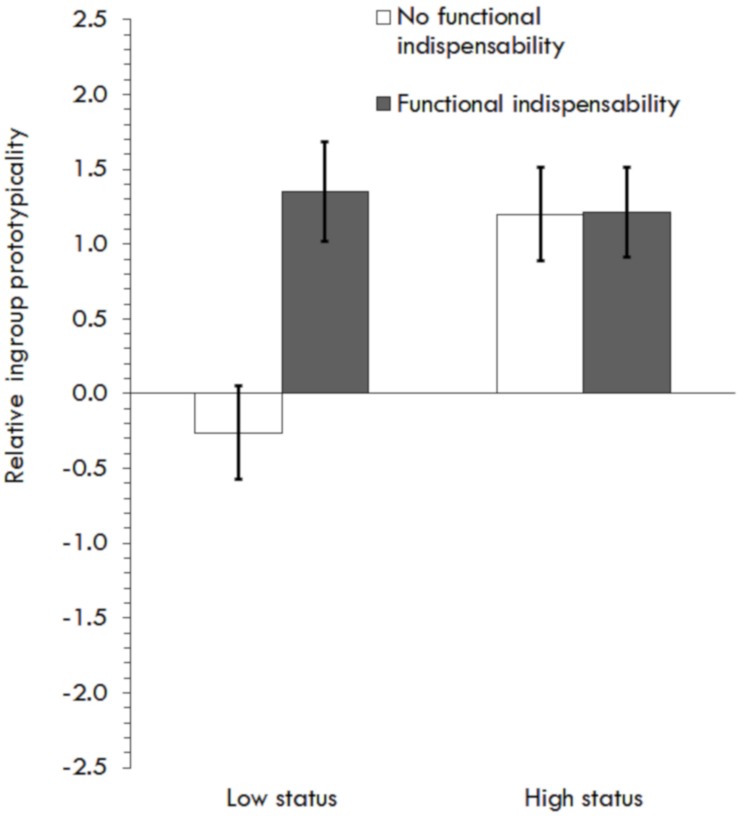
Effects of relative ingroup prototypicality depending on functional indispensability and status in Study 4.

The scenario was considered more realistic than unrealistic in general (*M* = 4.84, *SD* = 1.49) and, more importantly, no differences in realism were found between conditions (*p*’s > 0.25). Testing H1 as in Study 3, a 2 (Functional indispensability) × 2 (Status) GLM on relative ingroup prototypicality showed a main effect of indispensability, *F*(1,109) = 6.99, *p* = 0.01, η^2^*_*p*_* = 0.06, and status on relative ingroup prototypicality, *F*(1,109) = 4.98, *p* = 0.03, η^2^*_*p*_* = 0.04, qualified by an interaction, *F*(1,109) = 5.21, *p* = 0.02, η^2^*_*p*_* = 0.05. Simple paired comparisons showed differences in prototypicality between indispensability conditions only when status was low (*p* < 0.001) and differences in prototypicality between status conditions only in the no-indispensability condition (*p* < 0.001). Thus, indispensability impacted relative ingroup prototypicality when status was low (Figure 3 and Table 1).

H2 was tested as in Study 3 [customized model (Hayes, 2017), testing a serial mediation (Figure 1) with status moderating all paths except from identification to commitment]. Consistent with H1, there was an interaction between status and indispensability on prototypicality (Table 5). The effect was significant at low status, β = 0.44, *p* = 0.001, *CI* [0.18,0.69], but not at high status, β = 0.03, *p* = 0.79, *CI* [−0.21,0.28]. Post-merger identification was predicted by prototypicality and status, which did not interact. Commitment was only predicted by identification. The direct effect of prototypicality (i.e., bypassing identification) was not significant and did not interact with status. At low status, the predicted indirect effect was not significant but the direction was as expected, and for high status, the effect was also not significant but much smaller. The index of moderated mediation was not significant (Table 5). It is nevertheless worth noting that all paths of the sequential mediation alone were significant.

**TABLE 5:** Summary of sequential mediation, Study 4 (moderated by status).

**Outcome**												
		**Relative ingroup**				**Post-merger**				**Change**		
		**prototypicality (M1)**				**identification (M2)**				**commitment (Y)**		
**Antecedent**		**Coef.**	***SE***	***p***		**Coef.**	***SE***	***p***		**Coef.**	***SE***	***p***
Functional indispensability (X)	*a*_1_	0.23	0.09	0.01	*a*_2_	–0.003	0.06	0.87	*c′*	0.001	0.09	0.99
Status (W)		0.20	0.09	0.02		–0.27	0.09	0.004		0.10	0.10	0.28
Prototypicality		–	–	–	*d*_21_	0.24	0.10	0.01	*b*_1_	–0.09	0.10	0.36
Indispensability × Status		–0.20	0.09	0.02		0.07	0.10	0.46		–0.09	0.10	0.35
Prototypicality × Status		–	–	–		0.06	0.10	0.57		–0.02	0.10	0.81
Identification		–	–	–		–	–	–	*b*_2_	0.39	0.10	<0.001
Constant		0.01	0.09	0.92		–0.02	0.09	0.87		0.01	0.09	0.92
		*R*^2^ = 0.14				*R*^2^ = 0.11				*R*^2^ = 0.14		
		*F*(3,109) = 5.84, *p* = 0.001				*F*(5,107) = 2.55, *p* = 0.03				*F*(6,106) = 2.87, *p* = 0.01		
Change		*R*^2^ = 0.04			X × W	*R*^2^ = 0.005			X × W	*R*^2^ = 0.01		
		*F*(3,109) = 5.21, *p* = *0.02*				*F*(1,107) = 0.56, *p* = 0.46				*F*(1,106) = 0.88, *p* = 0.35		
					M1 × W	*R*^2^ = 0.003			M1 × W	*R*^2^ = 0.001		
						*F*(1,107) = 0.32, *p* = 0.57				*F*(1,106) = 0.06, *p* = 0.81		

**Conditional indirect effects**		**Coef.**				***Boot SE***		***Lower level 95% Boot CI***		***Upper level 95% Boot CI***		

At low status	*a_1_ b_1_*	–0.03				0.06		–0.14		0.10		
	*a_2_ b_2_*	–0.03				0.07		–0.16		0.11		
	*a_1_ d_21_ b_2_*	0.03				0.03		–0.03		0.09		
At high status	*a_1_ b_1_*	–0.004				0.02		–0.06		0.04		
	*a_2_ b_2_*	–0.03				0.05		–0.07		0.13		
	*a_1_ d_21_* b_2_	0.004				0.02		–0.04		0.03		
Index of moderated mediation	*a_1_ b_1_*	0.03				0.06		–0.11		0.15		
	*a_2_ b_2_*	0.06				0.08		–0.11		0.23		
	*a_1_ d_21_ b_2_*	–0.03				0.03		–0.10		0.03		

Additional GLMs of indispensability on post-merger identification, and on change commitment were conducted. Indispensability had no effect on post-merger identification, *F*(1,111) = 0.11, *p* = 0.74, η^2^*_*p*_* = 0.001, or change commitment, *F*(1,111) = 0.00, *p* = 0.99, η^2^*_*p*_* = 0.00. Given that previous theory links status asymmetry to group identification via prototypicality, this particular mediation was also tested (Model 4, Hayes, 2017). Indispensability was included as covariate. In sum, status positively predicted prototypicality, β = 0.20, *p* = 0.03, and prototypicality predicted identification, β = 0.22, *p* = 0.02. However, the direct effect of status on identification was also significant, β = −0.27, *p* = 0.004. The indirect effect was not significant, β = 0.05, *CI* [−0.01,0.11].

Taking into account the hypotheses’ testing, the improved manipulations in Study 4 led to stronger support for H1. However, although the direction was consistent with H2, the conditional mediation in the lower-status conditions was not significant. Also, the design did not allow for testing H3. Study 5 tested our hypotheses with a full design.

## Study 5

### Materials and Methods

Two hundred and thirty-seven participants completed the study, recruited in the same way as in Study 4. Data from 8 of them were excluded for participating in previous studies. The final sample included 229 participants (46 male, 183 female, 18–74 years old; *M* = 36, *SD* = 10.62). The study replicated Study 4, but including information processing: 2 (Status: High vs. low) × 2 (Indispensability: No indispensability vs. indispensability) × 2 (Information processing: Heuristic vs. systematic). Information processing was manipulated as in Studies 1–3 and indispensability and status were manipulated as in Study 4. Dependent measures were the same as in Study 4 (α = 0.81 for change commitment; α = 0.80 for group identification).

### Results and Discussion

**FIGURE 4:**
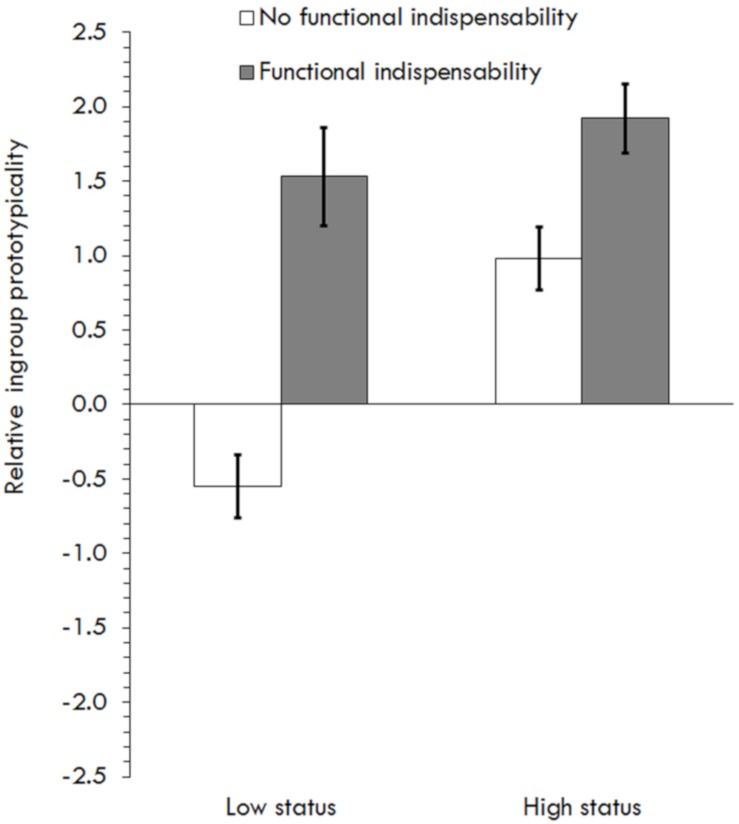
Effects of relative ingroup prototypicality depending on functional indispensability and status in Study 5.

Testing H1 and exploring H3, a 2 (Functional indispensability) × 2 (Status) × 2 (Information-processing) GLM on relative ingroup prototypicality showed a main effect of functional indispensability, *F*(1,221) = 50.04, *p* < 0.001, η^2^*_*p*_* = 0.19, and of status, *F*(1,221) = 16.36, *p* < 0.001, η^2^*_*p*_* = 0.07, qualified by an interaction, *F*(1,221) = 7.03, *p* = 0.01, η^2^*_*p*_* = 0.03. Simple main effects showed differences in prototypicality between indispensability conditions in both status conditions, but stronger for low status, *F*(1,221) = 47.26, *p* < 0.001, η^2^*_*p*_* = 0.18, than for high status, *F*(1,221) = 9.78, *p* = 0.002, η^2^*_*p*_* = 0.04. Simple main effects also showed differences in prototypicality between status conditions only when no indispensability information was given (*p* < 0.001) (Figure 4), supporting H1. However, results did not support H3, as no effects of information processing were found (*p*’s > 0.40) (Table 1).

Again, H2 was tested as previously [customized model (Hayes, 2017)]. Consistent with H1, there was an interaction between status and indispensability on prototypicality (Table 6). The effect was significant at both low status, β = 0.57, *p* < 0.001, *CI*_95_ [0.40,0.73], and high status, β = 0.26, *p* = 0.002, *CI*_95_ [0.10,0.42], but stronger for low status. There were no significant effects on post-merger identification. There was a significant effect of prototypicality and, as expected, of identification on commitment (Table 6). The predicted indirect effect was not significant, because relative ingroup prototypicality did not predict post-merger identification. An alternative mediation excluding post-merger identification (that is, the effect of indispensability on commitment mediated by prototypicality) was significant at low status, β = 0.08, *CI*_95_ [0.001,0.15], but not at high status, β = 0.04, *CI*_95_ [−0.02,0.11] (Table 6).

**TABLE 6:** Summary of sequential mediation, Study 5 (Figure 1 for paths).

**Outcome**												
		**Relative ingroup**				**Post-merger**				**Change**		
		**prototypicality (M1)**				**identification (M2)**				**commitment (Y)**		
**Antecedent**		**Coef.**	***SE***	***p***		**Coef.**	***SE***	***p***		**Coef.**	***SE***	***p***
Functional indispensability (X)	*a*_1_	0.41	0.06	<0.001	*a*_2_	0.08	0.07	0.31	*c′*	–0.11	0.06	0.08
Status (W)		0.24	0.06	<0.001		–0.07	0.07	0.32		–0.06	0.06	0.32
Prototypicality		–	–	–	*d*_21_	–0.01	0.08	0.85	*b*_1_	0.14	0.07	0.03
Indispensability × Status		–0.15	0.06	0.01		–0.02	0.07	0.82		0.05	0.06	0.43
Prototypicality × Status		–	–	–		0.01	0.08	0.85		0.01	0.07	0.87
Identification		–	–	–		–	–	–	*b*_2_	0.52	0.06	<0.001
Constant		–0.01	0.18	0.94		–0.01	0.21	0.95		0.10	0.18	0.57
Information Processing		0.01	0.12	0.94		0.01	0.13	0.96		–0.07	0.11	0.54
		*R*^2^ = 0.25				*R*^2^ = 0.05				*R*^2^ = 0.29		
		*F*(4,224) = 18.76, *p* < 0.001				*F*(6,222) = 0.40, *p* = 0.88				*F*(7,221) = 12.60, *p* < 0.001		
Change		*R*^2^ = 0.02			X × W	*R*^2^ = 0.0002			X × W	*R*^2^ = 0.002		
		*F*(1,224) = 6.92, *p* = 0.01				*F*(1,222) = 0.05, *p* = 0.82				*F*(1,221) = 0.263, *p* = 0.43		
					M1 × W	*R*^2^ = 0.0002			M1 × W	*R*^2^ = 0.0001		
						*F*(1,222) = 0.03, *p* = 0.85				*F*(1,221) = 0.03, *p* = 0.87		

**Conditional indirect effects**		**Coef.**				***Boot SE***		***Lower level 95% Boot CI***		***Upper level 95% Boot CI***		

At low status	*a_1_ b_1_*	0.08				0.04		0.001		0.15		
	*a_2_ b_2_*	0.05				0.06		–0.07		0.17		
	*a_1_ d_21_ b_2_*	–0.01				0.03		–0.08		0.06		
At high status	*a_1_ b_1_*	0.04				0.03		–0.02		0.11		
	*a_2_ b_2_*	0.03				0.05		–0.06		0.13		
	*a_1_ d_21_ b_2_*	–0.001				0.02		–0.04		0.03		
Index of moderated mediation	*a_1_ b_1_*	–0.03				0.05		–0.14		0.07		
	*a_2_ b_2_*	–0.02				0.08		–0.17		0.13		
	*a_1_ d_21_ b_2_*	0.01				0.04		–0.07		0.08		

Additional GLMs of indispensability on post-merger identification and change commitment were conducted. Functional indispensability had no effect on post-merger identification *F*(1,227) = 1.08, *p* = 0.30, η^2^*_*p*_* = 0.01, or change commitment, *F*(1,227) = 0.09, *p* = 0.77, η^2^*_*p*_* = 0.00. Status positively predicted prototypicality, β = 0.24, *p* < 0.001, but prototypicality did not predict identification, β = −0.01, *p* = 0.85. The indirect effect of status via prototypicality was, therefore, not significant, β = −0.004, *CI* [−0.04,0.03].

## General Discussion

Mergers involve working toward strategic goals that are often threatening to group members, especially from the partner having lower premerger status. Representativeness and identification with the merged group is often low for these partners (e.g., Giessner et al., 2012). This is an example of the so-called human factors that contribute to merger failure. We complement research on understanding merger failure with an approach on merger success, by suggesting opportunities for identification and commitment among members of low-status merging subgroups (merger partners). In the case of organizational mergers, there is a consensus in the literature that mergers imply particular challenges for employees, but even after Napier’s (Napier, 1989) concerns about the pre-merger phase reactions, most of the existent knowledge still relies on employees’ reactions in the post-merger phase (Teerikangas, 2010). Thus, we tested the effect of contributions of the pre-merger organization to the (future) success of the merger as an antecedent of relative ingroup prototypicality leading to post-merger identification and commitment to change, and examined these processes at an early stage, upon announcement.

We hypothesized that functional indispensability (instrumental contribution to a superordinate outcome; Guerra et al., 2015, 2016) impacted representativeness claims (relative ingroup prototypicality; Mummendey and Wenzel, 1999) (H1). Also, we examined whether indispensability leads to change commitment via relative ingroup prototypicality and post-merger identification (H2, Figure 1). Additionally, building on the fact that mergers are announced in a time-constrained and/or cognitively loaded environment, we explored whether the moderating effect of (systematic) information processing on the relation between merger patterns and relative ingroup prototypicality (Rosa et al., 2017) is also applicable to the relation between indispensability and relative ingroup prototypicality (H3). Five studies were conducted, each providing partial evidence for these hypotheses.

H1 was supported in all studies except Study 2: relative ingroup prototypicality increased given indispensability for the low-status merger partner (status was manipulated in Studies 3–5). More interestingly, in Studies 4 and 5, an interaction between status and indispensability indicated that the increase of relative ingroup prototypicality due to indispensability was more pronounced at lower status.

H2 was not fully supported, but partial evidence was found. More precisely, it was marginally supported in Studies 1 and 2. In Studies 3 and 4, the effect was not significant but still in the expected direction. Only in Study 5 was it clearly not supported, but a simpler mediation path was significant (indispensability predicting change commitment via relative ingroup prototypicality alone).

Information processing did not moderate the effect of indispensability on relative ingroup prototypicality (H3). Results showed that the effect of indispensability on relative ingroup prototypicality was exacerbated in systematic processing, but only in Study 2.

In order to summarize the findings, it is not advisable to conduct a mini meta-analysis on the complex analyses conducted (Goh et al., 2016). Nevertheless, we can conclude that no single study provided support for all hypotheses at once, but taken together, they show some evidence for, at least, H1 and H2, with intriguing implications. Theoretically, they provide insights for different literatures, and at an applied level, they offer insights for future research and for managing a structural intergroup change like a merger at its initial stages.

Merger situations are an important context to examine indispensability (Verkuyten et al., 2014), and our research provides promising steps in this direction. Functional indispensability might allow lower-status merger partners to construct identity fit by claiming representativeness. Also, we proposed that functional indispensability is an antecedent of relative ingroup prototypicality. By combining strategic (indispensability) and identity (post-merger identification) fit, managers can increase the likelihood that employees commit to changes implied by a merger. This is in line with recent approaches to mergers, counteracting the idea that mergers necessarily imply negative outcomes for employees (Teerikangas, 2010; Paustian-Underdahl et al., 2017). While mergers have been shown to imply identity discontinuity/loss, stress, and other negative challenges (e.g., Buono et al., 1985; Cartwright and Cooper, 1993; Giessner et al., 2016), employees can see the change in a positive light when it is perceived as a growth opportunity provided by the organization(s). Examples can be found among employees from companies in less economically advanced countries (Paustian-Underdahl et al., 2017), and when organizational fit is a source of growth opportunity (Teerikangas, 2010). As recent research indicates, we assume that functional indispensability can be related to growth opportunities (Wermser et al., 2018), especially for the lower-status merger partner.

This research offers important insights into lower-status groups’ prototypicality. Indeed, the most robust result of this research concerns the role of indispensability for relative ingroup prototypicality. Ingroup projection is uncommon and weak for low-status groups because status is a powerful prototypicality cue (e.g., Wenzel et al., 2007), but our studies show that functional indispensability might be a strong prototypicality cue regardless of status. We speculate that indispensability may offer opportunities for identity-management strategies (Ellemers and Van Rijswijk, 1997). Research with minority groups has shown that low-status groups can increase their relative ingroup prototypicality when they perceive that they contribute to the betterment of society, for instance when minority membership is self-selected based on strong (religious) beliefs (Alexandre et al., 2016a). In the case of mergers, functional indispensability can be one of such prototypicality cues, and it provides an important theoretical contribution to understand different constellations of status-asymmetric intergroup relations. It would be an interesting expansion to address in future research the role of indispensability in more complex instances of asymmetry, when status is not aligned with dominance [e.g., a more powerful partner acquiring a more prestigious/elite brand (van Knippenberg et al., 2002)].

Also, indispensability-fueled relative ingroup prototypicality might lead to change commitment via post-merger identification. Previous research showed that pre-merger status and, more importantly, representativeness predicted post-merger identification and bias toward the merger partner (Boen et al., 2010) but our research provides a more positive outlook on mergers by associating representativeness with change commitment. Nevertheless, the fact that the predicted sequential mediation was not fully supported across all studies raises questions for further inquiry. We can deduce that there is more in terms of consequences of relative ingroup prototypicality that we did not address in this research. For instance, the finding that indispensability led to change commitment without being mediated by post-merger identification (Study 5) suggests that other mechanisms may be needed to capture a more complete picture. For example, Blader and Yu (2017) proposed that intragroup respect is a source of social worth complementary to status, which meets affiliation motives and leads to commitment to contribute toward the group goals, especially when the group is under threat. Respect can be related to justice concerns, which are an important antecedent of post-merger identification (Giessner et al., 2012), but seldomly studied from an intergroup perspective. Research on intergroup equality-based respect has found that perceived respect from outgroups promotes intergroup harmony (Simon et al., 2015), which might be good for merger integration. On the other hand, respect at an intergroup level can lead to conformity, at least in early intergroup interactions (Renger et al., 2019). Examining the interplay between status, indispensability and respect on identification, relative ingroup prototypicality and commitment could inform a more comprehensive approach to reactions to a merger announcement.

We attempted to combine social identity processes with motivated cognition. In Study 2, relative ingroup prototypicality was higher under systematic processing in the indispensability condition, corroborating previous findings with merger patterns (Rosa et al., 2017). Indispensability perceptions might produce dissonance with status-related prototypicality cues that categorize low-status groups as inferior. Therefore, employees from the lower pre-merger status partner might need cognitive/motivational resources to fully process the information and form judgments, allowing them a better adaptation to the changes. However, information processing did not play a role in any of the other studies. Thus, the role of information processing in the effects of indispensability information is left for future inquiries. Study 1 was conducted using a real situation but not targeting employees, and students were generally happy about the prospect of a merger. On the other hand, Studies 2–5 focused on a working population but used a scenario. Scenarios are very helpful for studying phenomena without interference from external factors such as the history of a given group, are commonly used to study mergers (Stahl and Pablo, 2003; Giessner et al., 2006; Thorbjørnsen and Dahlén, 2011), and we considered it as the logical first step to test a clean causal effect. For instance, Robinson and Clore (2001) compared vignette approaches (simulations, asking participants to estimate their likely reactions) with online appraisals on its capacity to capture emotional experiences. Participants were able to accurately estimate their likely reactions in simulated approaches and the authors concluded that “vignette methodologies can play a useful role in theory construction” (*p*. 1520). However, our merger scenario required initial involvement from participants, and for that reason, the processing manipulation was introduced only after the scenario. Still, mergers are an intergroup context that is inherently insecure/defensive (Ivancevich et al., 1987; Buono and Bowditch, 1989; Rentsch and Schneider, 1991; Cartwright and Cooper, 1993; van Dick et al., 2006) and such insecure situations may trigger defense motives when processing information. In such cases, both heuristic and systematic processing can be used, on the basis of whichever of these best fulfills the defense goal (Giner-Sorolla and Chaiken, 1997). Thus, processing information about a merger includes not only an intellectual task, but also a tough emotional management, dealing with issues of meaning, trust, sacrifice, fears about the future, etc. (e.g., Kotter, 2012). We are not sure whether the scenario, though considered realistic by the participants, provoked enough defensive responses compared with a real situation, especially considering that we captured very initial reactions. For instance, research conducted on real mergers has shown that given little information is provided at time of the merger announcement, employees end up engaging in rumors (Stahl and Sitkin, 2010) and opinions intensify as discussions with colleagues and gossiping increase (Marmenout, 2010, 2011). Our design did not allow to capture these important elements. While information processing previously showed a moderating role in the effect of merger patterns on relative ingroup prototypicality (Rosa et al., 2017), it was not the case with functional indispensability, probably because for manipulating merger patterns, the scenario can be more straightforwardly interpretable. As cues for future research addressing this question, we wonder whether such manipulation of time pressure at the stage of judgment formation (vs. reporting) would have provided support for H3, or if a more specific manipulation of information processing, such as cognitive load, or even accuracy motivation could be stronger in this context, compared with time pressure. Future research should try to study the phenomenon in organizational contexts to ascertain the generalizability of our findings. For instance, it would be valuable to conduct a study having participants from the same group/organization, thus not requiring an initial learning period to emerge in the fictitious groups depicted in the scenario. This would allow for a more sophisticated test of our hypotheses, even if not addressing a real merger situation but a hypothetical one, within a previously known ingroup.

Furthermore, we can speculate whether this functional approach would be generalizable from this context to more societal contexts, for which instrumentality is a less viable prototypicality cue. For other minorities (e.g., ethnic, gender), functional indispensability alone might not be enough to improve identity-motivated judgments. Also, just as social indispensability is highly applicable within sports teams (e.g., Hertel et al., 2008), we speculate that functional indispensability effects on representativeness could be generalizable to other contexts not necessarily involving change/restructuring, such as intergroup sports competitions.

In sum, this research offers initial, yet important insights into the constellation of challenges in mergers as seen from the perspective of social identity and motivated cognition, and opens important avenues to the study of the human side of mergers, from the moment of their announcement.

## Data Availability Statement

The datasets generated for this study can be found in the Open Science Framework, https://osf.io/6wfpx/.

## Ethics Statement

The studies involving human participants were reviewed and approved by the Erasmus Research Institute of Management (Erasmus University Rotterdam), and specific approval for Study 2 was granted from University of Salzburg. The participants digitally provided their informed consent to participate in this study.

## Author Contributions

MR contributed with the conception, design, statistical analyses, writing the first draft, supervising data collection, and coordinating the overall research. SG, RG, SW, and EC contributed substantially to the conception and design of the work, interpretation of findings and critical revision of contents. A-MK and KV contributed to the design, data collection, and interpretation of Study 2. All authors contributed to manuscript revision, and read and approved the submitted version.

## Conflict of Interest

The authors declare that the research was conducted in the absence of any commercial or financial relationships that could be construed as a potential conflict of interest.

## Footnotes

1 Although mergers and acquisitions have several differences, we use the general term mergers without distinguishing between a merger and an acquisition.

2 Participation from students involved a 30-min timeslot. Thus, we presented a bigger questionnaire, measuring other accessory variables that could be used as potential controls, but we did not use them in the analyses. These measures were: attitudes toward ingroup and outgroup, status legitimacy, ingroup pride, attitude certainty about the alliance, ingroup identification, social influence/persuasion, and some items from an attempted relative ingroup prototypicality verbal scale.

3 For exploratory reasons, we also included two single items at the end of the questionnaire that made reference to status and indispensability. However, as these items were new and preliminary, we did not use them as manipulation checks but relied instead on the pilot for the validity of our manipulations.

## Abbreviations

SC: superordinate category.
